# Effects of immersive virtual therapy on psychological and physical wellbeing in women with breast cancer at different stages of disease and treatment conditions: an experimental comparative study

**DOI:** 10.3389/fonc.2025.1581604

**Published:** 2025-08-04

**Authors:** Oliver Czech, Rafal Matkowski, Jakub Skórniak, Iwona Malicka

**Affiliations:** ^1^ Department of Physiotherapy, Wroclaw University of Health and Sport Sciences, Wroclaw, Poland; ^2^ Lower Silesian Oncology, Pulmonology and Hematology Center, Wroclaw, Poland; ^3^ Department of Oncology, Faculty of Medicine, Wroclaw Medical University, Wroclaw, Poland

**Keywords:** breast cancer, physical wellbeing, mental wellbeing, rehabilitation, virtual reality, modern approach

## Abstract

**Background:**

Breast cancer (BC) is the most common cancer worldwide, with rising incidence and complex treatment challanges. Patients often experience physical and psychological symptoms that negatively affect their quality of life. Virtual Reality (VR) has shown promise as a supportive, non-pharmacological intervention by reducing anxiety, depression, pain and aiding physical rehabilitation in cancer care.

**Objective:**

This study evaluated the impact of immersive VR therapy in improving the psychological and physical wellbeing of women with BC at different stages of treatment and compared outcomes across treatment groups.

**Methods:**

Fifty-six women were recruited at the Lower Silesian Oncology, Pulmonology and Hematology Center in Wroclaw, Poland, and divided into four equal groups (n = 14) based on their treatment stage: recently diagnosed before treatment (diagnosis group), in the perioperative period (surgery group), undergoing radiotherapy (radiotherapy group), and post-treatment (healed group). All participants completed ten VR therapy sessions involving therapeutic tasks in a virtual garden. Outcome measures included anxiety (State-Trait Anxiety Inventory), depression (Beck Depression Inventory), coping strategies (Mini-Mental Adjustment to Cancer), physical activity (International Physical Activity Questionnaire), and sleep quality (Pittsburgh Sleep Quality Index). Repeated measures ANOVA was used to) assess changes over time.

**Results:**

Anxiety (F = 15.82, η_p_² = 0.23, p < 0.001), depression (F = 32.48, η_p_² = 0.38, p < 0.001), coping strategies (F = 4.78, η_p_² = 0.08, p = 0.03), physical activity (F = 6.48, η_p_² = 0.11, p = 0.01, and F = 6.97, η_p_² = 0.12, p = 0.01), and sleep quality (F = 9.36, η_p_² = 0.15, p < 0.01) improved significantly. However, no significant differences were found between treatment groups.

**Conclusions:**

Immersive VR therapy effectively supports both mental and physical wellbeing in woman with BC, regardless of treatment stage. These findings suggest VR is a versatile and valuable tool for supportive care in oncology. Future studies should explore it’s use in broader clinical settings, including outpatient and home-based care.

## Introduction

Breast cancer (BC) is the most frequently diagnosed malignancy and the second most frequent cause of cancer-related death in women globally. According to GLOBOCAN 2020 data, approx. 2.3 million new cases were reported worldwide, accounting for about 12% of all cancer diagnoses and leading to over 685,000 deaths annually. In Europe alone, BC represents the most common malignancy in woman, with one in eight women expected to develop the disease during their lifetime (1). While advances in early detection, diagnostics, and treatment—such as surgery, chemotherapy, radiotherapy, hormone therapy, and targeted therapies—have led to a measurable decline in mortality rates, the number of survivors dealing with the long-term physical and psychological consequences of the disease continues to rise (2).

The consequences are far-reaching. Physical complications such as pain, reduced range of motion, fatigue and cognitive impairments often co-occur, with psychological symptoms like anxiety, depression, and distress. These impairments negatively affect daily functioning, social relationships, and occupational performance, thereby reducing overall quality of life (3, 4). Also, physical and psychological symptoms can affect each other—for example, ongoing pain can make depression worse—which can lead to reduced daily functioning and higher healthcare needs (5, 6). Inadequately managed psychological distress is associated with poorer treatment adherence, elevated costs, and increased risk of disease relapse and mortality (7, 8). As such, these factors can, individually or generally, influence patient quality of life, deteriorate general wellbeing and overall health (2, 3, 5).

To address these complex needs, oncology care is increasingly incorporating integrative and supportive interventions that go beyond pharmacological solutions. One such complementary and integrative technology for cancer prehabilitation and rehabilitation is Virtual Reality (VR),which creates immersive, interactive environments using audio-visual and sensory stimuli that can transport patients out of stressful clinical settings, reduce perception of pain and anxiety, and motivate to increase physical activity (9–12). Due to the high immersion and interaction caused by head-mounted displays, motion-tracking equipment, and controllers, VR allows the patient to be isolated from the medical environment (9, 13). Thus, VR is often used as a distraction therapy during painful or unpleasant interventions, as a motivational therapy supporting treatment demanding high participation and interaction, and for psychical therapy (13–15). The technology can also enable real-time monitoring and adaptive feedback, supporting personalized therapy (16). Additionally, VR use is relatively safe and straightforward, allowing medical staff and medical facilities to use it more often to manage BC patient’s physical and psychological symptoms (17, 18). These features make VR a potentially valuable adjunct in managing both physical and psychological symptoms in patients with BC (12, 16, 19).

## Materials and methods

### Ethical considerations

The Ethical Committee of the University School of Physical Education in Wroclaw (approval number 18/2019) and the Institutional Review Board of the Lower Silesian Center of Oncology, Pulmonology, and Hematology in Wroclaw approved the study, which was registered with the Australian New Zealand Clinical Trials Registry (ACTRN12623000085673).

### Participants

Participants were recruited at the Breast Unit of the Lower Silesian Oncology, Pulmonology, and Hematology Center in Wroclaw, Poland. The research was conducted from June 2021 to October 2023. Inclusion criteria were a cancer diagnosis, with or without treatment commencement, and informed consent to participate in the study. The study also enrolled only participants with adequate cognitive function, allowing comprehension of instructions and independent use of VR equipment, with stable general health condition, allowing participation in repeated VR sessions lasting approximately 15–20 minutes and with sufficient visual and auditory function (with or without correction), enabling engagement with VR audiovisual content. Exclusion criteria included patients with cognitive impairments, as documented in their medical records, or those who had received psychiatric treatment in the past or during the study period. Also patients with epilepsy, motion sickness, vestibular dysfunction, visual or hearing impairments, treated with drugs, which could affect perception, and other patients which could not complete the VR sessions for logistical, medical or organizational reasons has been excluded from participation. All patients, present in the hospital during the research, who met the described criteria has been invited to participate in the study.

After initial screening, patients were stratified based on treatment stage at the time of recruitment: diagnosed with cancer, with no treatment started (diagnosis group - DG), during hospitalisation for surgical cancer treatment (surgical group - SG), during a radiotherapy treatment cycle (radiotherapy group - RG), and cancer treatment completed (healed group - HG).

Within each stratum, all participants were assigned to the experimental group, as the primary study goal was to evaluate the effectiveness of the intervention at different treatment stages. There was no randomization within strata since the objective was to assess intervention efficacy at specific phases of treatment. Thus, patients in each stratum received the same experimental treatment and were evaluated for method effectiveness relative to their stage in the treatment process.

### Procedure

The outcome measures were repeated twice, including baseline pre-intervention (T0) and post-intervention, 2 weeks after baseline (T1). Medical staff, trained by the VR system inventor and producer (VRTier One, Stanowice, Poland), monitored the sessions, with each lasting approximately 15 minutes (min 13:50 –max 17:50 for therapeutic sessions), and all patients participating in ten sessions, including one for demonstration and one for summary. Patients were immersed in a virtual garden featuring therapeutic audio stimuli, with the garden design evolving from a dull, colourless landscape that progressively gained colour, vitality, and beauty by the final session. The audio commentary, crafted as a comprehensive therapeutic cycle, varied between sessions. Each session also included physical tasks such as creating a virtual mandala (unique for each session), tending the garden, and breathing exercises.

The VR setup consisted of VR goggles and a controller (manipulator) connected to a personal computer (PC), with the immersive experience provided by the head-mounted display delivering intense visual, auditory, and kinesthetic stimuli. The sessions were intentionally designed to be calming and mood-enhancing. Prior research on the VR TierOne device found that it helped patients tap into their psychological resources and encouraged more active participation in rehabilitation (20–22).

The virtual garden used symbols and metaphors aligned with the Ericksonian psychotherapeutic approach, with the “Garden of Revival”, representing the patient’s health, a particularly significant aspect. As the patient cultivated the garden, the virtual environment came alive, symbolising the healing process. This metaphorical approach reduces patient resistance by not directly addressing their health status or life situation. Instead, it depicts a parallel process unfolding before their eyes. Employing the Ericksonian method encourages self-healing processes, enhancing the therapeutic effects of the sessions (20–22).

### Measures

All participants completed a sociodemographic questionnaire that gathered basic personal information, including details about their education level, living situation, financial status, and marital status (measured on a 4-point Likert scale). In addition, medical data was collected, including information on estrogen and progesterone receptor expression, human epidermal growth fact 2 (HER2)-positive BC, a Ki-67 proliferation index greater than 25%, and clinical staging (cTNM). Participants also completed five standardized questionnaires assessing their levels of anxiety and depression, coping strategies related to the disease, physical activity (PA), and sleep quality.

### Spielberger state-trait anxiety inventory

The Speilberger State-Trait Anxiety Inventory (STAI) is a self-reported 40-item questionnaire designed to assess anxiety state and traits, with 20 items dedicated to each. The current study only used the State Anxiety subscale (STAI-X1), which is highly sensitive and effectively captures fluctuations in anxiety levels over short intervals. The questions focus on the individual’s current emotional state (e.g., tension, nervousness, and worry), and responses are rated on a 4-point scale, with higher scores indicating more severe anxiety. Originally developed to measure an individual’s predisposition to anxiety, the STAI is particularly relevant in cancer treatment contexts, where anxiety levels are often elevated. In this study, the tool evaluated how a BC diagnosis impacts anxiety as a chronic condition. Cronbach alpha for STAI was indicated as 0.92 (23).

### Beck depression inventory

The Beck Depression Index (BDI) is a well-established psychometric tool used to assess depression severity. Known for its high reliability, it can effectively distinguish between depressed and non-depressed individuals and has strong concurrent, content, and structural validity. Frequently used in general population screenings and studies involving patients with various health conditions, the BDI comprises 21 items covering cognitive-affective and somatic symptoms of mood disorders, with a total score of 0–10 considered normal, 11–26 indicating mild depressive symptoms, and above 27 suggesting clinical depression. The tool determined if emotional responses to a BC diagnosis contribute to the development of depressive states. Cronbach alpha for BDI was indicated as 0.9 (24).

### Mini-mental adjustment to cancer scale

The Mini-Mental Adjustment to Cancer Scale (Mini-MAC) is a 29-item questionnaire used to assess an individual’s emotional and cognitive response to a cancer diagnosis by evaluating coping strategies on a 4-point Likert scale. The four coping style categories include anxious preoccupation, fighting spirit, helplessness-hopelessness, and positive redefinition. The total score ranges from 7 to 28 points and is further divided into destructive (anxious preoccupation and helplessness-hopelessness) and constructive (fighting spirit and positive redefinition) coping styles. In summary, the scale helps assess how patients cope with cancer and how their coping mechanisms might influence their psychological wellbeing. Cronbach alpha for Mini-MAC was indicated as 0.75 (25).

### International physical activity questionnaire

The International Physical Activity Questionnaire (IPAQ) measures PA and sedentary behaviour in adults based on responses to seven questions covering one week and allows for a comprehensive assessment of an individual’s PA levels across various domains (26). The questions address five domains of activity: occupational, transportation, housework and caregiving, recreational or sports activities, and time spent sitting. The questionnaire calculates the total amount of PA in metabolic equivalent (MET)-minutes per week, and participants are categorised into three activity levels: insufficient (less than 600 MET-min/week), sufficient (600–1500 or 600–3000 MET-min/week), or high (more than 1500 or 3000 MET-min/week). Cronbach alpha for IPAQ was indicated as 0.8.

### Pittsburgh sleep quality index

Evaluation of participants’ sleep patterns and the impact of cancer on sleep quality used the Pittsburgh Sleep Quality Index (PSQI), a 19-item questionnaire that assesses sleep quality and patterns over the past month and is widely used in clinical and research settings due to its proven reliability (27). The index evaluates sleep quality across seven categories: (1) subjective quality of sleep, (2) sleep latency, (3) sleep duration, (4) habitual sleep efficiency, (5) sleep disturbances, (6) use of sleep medication, and (7) daytime dysfunction. Each category uses a 4-point scale, with the scores summed to create a global index ranging from 0 to 21. Higher scores indicate lower sleep quality. Cronbach alpha for PSQI was indicated as 0.73

### Data analysis

All statistical analyses employed JASP version 0.19.1 (University of Amsterdam, The Netherlands). Data distribution analysis was performed using the Shapiro-Wilk test, baseline between-group data with continuous variables were compared using analysis of variance (ANOVA), and the Chi-squared test assessed categorical variables. The effect of the intervention between the strata was calculated using repeated measures ANOVA (RM-ANOVA) with Bonferronis’ *post hoc* analysis. This approach aimed to isolate the impact of the treatment at each stage of the therapeutic process. All analyses used p < 0.05 to indicate statistical significance.

GPower 3.1.9.2 software (G*Power, Heinrich-Heine University Düsseldorf, Düsseldorf, Germany) assisted sample calculation using the parameters α = 0.05, f = 0.80, and 95% power. The output of this calculation suggested 12 samples per group. Due to an estimated 15% dropout rate, the sample size was set at 14 per group (28).

## Results

The study involved 56 women (n =14 per group) diagnosed with BC. All the invited patients accepted the invitation to the study and gave their written consent to participate. **Table 1** lists the quantities of patient cancer staging for each group according to cTNM classification.

**Table 1:** Cancer stage characteristics.

Cancer stage *n* (%)	DG	SG	RG	HG	*p*-Value *
cTis (DCIS)	1 (7)	0 (0)	1 (7)	1 (7)	0.51
cT1N0M0	3 (21)	6 (43)	7 (51)	5 (37)	
cT1N1M0	1 (7)	2 (14)	0 (0)	0 (0)	
cT1N2M0	1 (7)	0 (0)	0 (0)	0 (0)	
cT2N0M0	4 (30)	4 (29)	1 (7)	2 (14)	
cT2N1M0	2 (14)	1 (7)	0 (0)	3 (21)	
cT2N2M0	0 (0)	0 (0)	1 (7)	0 (0)	
cT3N0M0	2 (14)	1 (7)	1 (7)	0 (0)	
cT3N1M0	0 (0)	0 (0)	0 (0)	1 (7)	
cT3N2M0	0 (0)	0 (0)	1 (7)	0 (0)	
cT3N3M0	0 (0)	0 (0)	0 (0)	1 (7)	
cT4N1M0	0 (0)	0 (0)	1 (7)	0 (0)	
cT4N1M1	0 (0)	0 (0)	1 (7)	0 (0)	
cT4N2M0	0 (0)	0 (0)	0 (0)	1 (7)	

*Chi-squared test.

Analyses of demographic data (**Table 2**) revealed no statistically significant differences between the study groups at baseline, except for patient age (p < 0.001, *post hoc*: HG vs DG, p = 0.004; HG vs SG, p = 0.03; HG vs RG, p = 0.001). Clinical data analysis revealed no differences between the study groups at baseline, except for progesterone (p = 0.02, *post hoc*: HG vs SG, p = 0.01) and estrogen receptor expression (p = 0.04, *post hoc*: HG vs SG, p = 0.02). Analyses of measured outcomes (**Table 3**) revealed no differences between the study groups at baseline, except for STAI (p < 0.01, *post hoc*: p > 0.05 for all baseline comparisons). Also, the T1 data only showed statistical significance for STAI results (p < 0.01, *post hoc*: DG vs HG, p < 0.01).

**Table 2:** Baseline demographic characteristics.

Variable	DG	SG	RG	HG	*p*-Value *
*n*	14	14	14	14	–
Age, [years]; mean (SD)	54.65 (13.13)	57.81 (13.93)	52.93 (12.92)	71.27 (7.61)	<0.001†
BMI, [kg/m^2^]; mean (SD)	27.03 (6.86)	26.67 (6.18)	25.43 (4.87)	28.61 (3.00)	0.49
Education, *n* (%)					0.48
Primary/Vocational	3 (21)	3 (22)	1 (7)	1 (7)	
Secondary	1 (7)	2 (14)	5 (36)	6 (43)	
Incomplete Higher Education	1 (7)	2 (14)	2 (14)	2 (14)	
Higher Education	9 (65)	7 (50)	6 (43)	5 (36)	
Marital status, *n* (%)					0.30
Single	3 (22)	1 (7)	1 (7)	2 (14)	
Married	9 (64)	9 (64)	6 (43)	4 (29)	
Cohabiting	1 (7)	3 (21)	2 (14)	1 (7)	
Widowed	0 (0)	1 (7)	3 (22)	4 (29)	
Divorced	1 (7)	0 (0)	2 (14)	3 (21)	
Fertility, *n* (%)					0.92
0	4 (29)	3 (21)	3 (21)	3 (21)	
1	2 (14)	2 (14)	4 (29)	4 (29)	
2	6 (43)	5 (36)	6 (43)	5 (36)	
3 or more	2 (14)	4 (29)	1 (7)	2 (14)	
Material status, *n* (%)					0.23
Bad	1 (7)	0 (0)	0 (0)	0 (0)	
Average	3 (22)	4 (29)	7 (50)	7 (50)	
Good	8 (57)	9 (64)	4 (29)	3 (21)	
Very Good	2 (14)	1 (7)	3 (21)	4 (29)	

†, statistical significance; SD, standard deviation; BMI, body mass index; * - analysis of variance or Chi-squared test as appropriate.

**Table 3:** Mean values of measured outcomes.

Variable	Timepoint	DG	SG	RG	HG	*p*-Value
STAI	Pre	52.71 (8.91)	44.14 (10.87)	47.00 (6.53)	39.57 (10.50)	<0.01†
	Post	47.36 (9.20)	37.71 (9.24)	41.43 (9.37)	32.79 (11.89)	<0.01†
BDI	Pre	13.29 (7.83)	9.64 (5.49)	12.79 (5.59)	10.64 (5.20)	0.35
	Post	8.64 (6.59)	5.00 (3.40)	9.29 (6.46)	8.14 (5.76)	0.21
Mini-MAC						
CS	Pre	42.86 (5.42)	43.43 (6.07)	41.93 (4.14)	47.00 (3.70)	0.06
	Post	43.71 (4.68)	43.43 (6.53)	42.64 (4.88)	47.43 (4.86)	0.09
DS	Pre	33.00 (5.79)	29.79 (7.90)	28.21 (5.10)	26.64 (6.30)	0.07
	Post	29.50 (7.24)	27.79 (6.04)	27.79 (6.33)	25.00 (5.44)	0.31
IPAQ						
Sedentary	Pre	1771.43 (1143.83)	1153.57 (1160.98)	1146.43 (784.09)	975.00 (1271.09)	0.25
	Post	1314.29 (875.40)	653.57 (674.95)	996.43 (709.95)	771.43 (712.99)	0.11
Walking	Pre	957.00 (914.68)	1225.71 (2866.80)	1105.50 (1669.03)	1374.21 (892.51)	0.94
	Post	1283.46 (965.02)	1780.82 (2850.84)	1112.57 (1682.71)	1493.25 (992.32)	0.78
Moderate	Pre	357.14 (672.35)	651.43 (1055.09)	241.43 (321.01)	617.14 (793.00)	0.41
	Post	1048.57 (1292.27)	325.71 (719.12)	328.57 (639.46)	1152.86 (1594.79)	0.10
Intense	Pre	105.71 (289.98)	905.71 (2147.29)	402.86 (907.33)	571.43 (882.43)	0.40
	Post	154.29 (272.59)	548.57 (2052.57)	51.43 (192.43)	1177.14 (2683.27)	0.30
Overall	Pre	1419.86 (1004.28)	2782.86 (4031.67)	1749.79 (2071.04)	2562.79 (1998.36)	0.44
	Post	2486.32 (1775.11)	2655.11 (4347.72)	1492.57 (1838.66)	3823.25 (4627.13)	0.36
PSQI						
Sleeping time	Pre	6.29 (1.72)	7.11 (1.44)	6.96 (1.64)	6.98 (1.09)	0.46
	Post	6.50 (1.35)	7.70 (1.39)	7.39 (1.52)	7.18 (1.35)	0.15
Sleep efficiency	Pre	77.90 (14.51)	83.90 (11.51)	81.78 (16.04)	82.36 (11.79)	0.69
	Post	82.84 (10.87)	87.07 (8.74)	85.10 (15.25)	84.36 (10.10)	0.81
Overall	Pre	7.86 (3.82)	6.64 (3.67)	7.79 (3.81)	6.36 (2.74)	0.58
	Post	6.57 (3.03)	4.79 (2.42)	5.64 (2.44)	5.71 (2.64)	0.37

†, statistical significance; DG, diagnosis group; HG, healed group; SG, surgery group; RG, radiotherapy group; STAI, Spielberger State-Trait Anxiety Inventory; BDI, Beck Depression Inventory; Mini-MAC, Mental Adjustment to Cancer Scale; IPAQ, International Physical Activity Questionnaire; PSQI, Pittsburgh Sleep Quality Index; CS, constructive style of coping; DS, destructive style of coping. Values are presented as mean and standard deviation.

Results of RM-ANOVA revealed statistically significant effects of time for STAI (F = 15.82, η_p_² = 0.23, p < 0.001), BDI (F = 32.48, η_p_² = 0.38, p < 0.001), destructive style of coping (F = 4.78, η_p_² = 0.08, p = 0.03), sedentary behaviour (F = 6.48, η_p_² = 0.11, p = 0.01) walking activity (F = 6.97, η_p_² = 0.12, p = 0.01), and overall PSQI score (F = 9.36, η_p_² = 0.15, p < 0.01). The RM-ANOVA results indicated no effects (p > 0.05) of time × group interaction, except for moderate PA (F = 2.91, η_p_² = 0.14, p = 0.04) (**Table 4**). However, *post hoc* analysis confirmed that this effect was not statistically significant (p > 0.05 for all between-group comparisons).

**Table 4:** Repeated measures analysis of variance results.

Variable	Effect	Mean square	F	η_p_²	*p*-Value
STAI	time	1020.04	15.82	0.23	<0.001†
	time × group	3.25	0.05	0.01	0.99
BDI	time	408.89	32.48	0.38	<0.001†
	time × group	7.46	0.59	0.03	0.62
Mini-MAC					
CS	time	7.00	0.68	0.01	0.41
	time × group	1.00	0.10	0.01	0.96
DS	time	100.32	4.78	0.08	0.03†
	time × group	11.20	0.53	0.03	0.66
IPAQ					
Sedentary	time	3.10×10^7^	6.48	0.11	0.01†
	time × group	2.18×10^5^	0.47	0.03	0.71
Walking	time	1.78×10^7^	6.97	0.12	0.01†
	time × group	4.09×10^5^	1.60	0.09	0.20
Moderate	time	1.71×10^7^	3.37	0.06	0.07
	time × group	1.48×10^7^	2.91	0.14	0.04†
Intense	time	5.16×10^3^	0.01	9.89×10^-5^	0.94
	time × group	1.45×10^7^	1.44	0.08	0.24
Overall	time	6.60×10^7^	2.66	0.05	0.11
	time × group	4.35×10^7^	1.75	0.09	0.17
PSQI					
Sleeping time	time	3.57	3.14	0.06	0.08
	time × group	0.25	0.22	0.01	0.89
Sleep efficiency	time	315.47	2.03	0.04	0.16
	time × group	10.18	0.07	0.01	0.98
Overall	time	61.51	9.36	0.15	<0.01†
	time × group	3.08	0.47	0.03	0.71

†, statistical significance; η_p_², eta partial square; STAI, Spielberger State-Trait Anxiety Inventory; BDI, Beck Depression Inventory; Mini-MAC, Mental Adjustment to Cancer Scale; IPAQ, International Physical Activity Questionnaire; PSQI, Pittsburgh Sleep Quality Index; CS, constructive style of coping; DS, destructive style of coping. Values are presented as mean and standard deviation.

## Discussion

Due to the significant number of factors influencing the psychophysical wellbeing of BC patients during diagnosis and treatment, it seems justified to determine whether modern forms of supporting oncological treatment are equally effective at every stage of the disease. The results of reviews and meta-analyses are consistent in their conclusions, confirming the relationship between VR and improved psychophysical outcomes at various stages of the disease course (29–31).

BC survivors experience more cognitive, sexual, fatigue, and anxiety issues than those without BC, particularly when more aggressive treatments are involved (32, 33). After surgery, patients often face significant psychological challenges, with coping strategies and resilience playing a key role in recovery, as seen in studies by Eker et al. Radiotherapy patients tend to use active coping but report a lower quality of life compared to healthy individuals, suggesting the need for tailored interventions. Long-term BC survivors, especially those without partners or children, may experience a decline in psychosocial wellbeing over time. Additionally, chemotherapy patients are at high risk for fatigue, particularly those with multiple cycles or particular genetic markers, making their psychological recovery more complex (34–39).

The complexity of cancer, the availability of very diverse forms of treatment, as well as individual predispositions and the patient’s environment mean that the results of similar studies may be inconsistent. It is still difficult to draw uniform conclusions that clearly confirm the effectiveness of VR in the treatment of symptoms developed during the presence of cancer. Contemporary reviews and meta-analyses highlight the large differences in the quality of the scientific papers presented, the large differences in the technology used, and the significant differences in the study design itself (30, 40–43). A scoping review by Su et al. (44) concludes, several VR studies are based on commercially available video games, rather than targeted therapeutical tools. This also could be a factor causing the inconsistent effectiveness of VR in research. It is worth noting, VR TierOne is a therapeutical tool invented, created and designed for patients with various diseases, focused on their psychical wellbeing and implementing upper limb tasks and breathing exercise for physical condition improvement.

Although various factors affecting quality of life and psychological wellbeing are described, the sum of these stimuli results in significant deterioration of patient psychological parameters. The study shows that the demographic, clinical and psychological profile of oncology patients is relatively homogeneous. Furthermore, the analysis found significant differences in participant age and confirmed that cancer survivors had a significantly higher average age than all other groups, which seems justified since cancer treatment is a lengthy process. Regardless of the treatment stage, the frequency of anxiety and depression symptoms, ways of coping with the disease, levels of PA, and sleep quality seem to be very similar.

All examined parameters are indicative of quality of life and general wellbeing. Thus, the obtained results confirm that VR can improve the quality of life and psychophysical wellbeing of patients, positively influencing the symptoms of anxiety and depression, ways of coping with cancer and low-intensity PA, and improving the overall quality of sleep, regardless of disease stage. As shown in the results section, no significant effects of time × group interaction were found. However, the analysis uncovered marked effects of time for STAI, BDI, destructive coping style, sedentary behaviour, walking activity and overall PSQI score. These results confirm that VR is effective in treating anxiety and depression and improving coping style, PA, and sleep quality, regardless of cancer or treatment stage.

Unfortunately, the positive effects described seem insufficient for some outcomes in the population. For STAI results, 0–9 points are considered no or normal anxiety, 10–18 indicates mild anxiety, 19–29 moderate to severe anxiety, and 30+ equates to severe anxiety. Despite a mean improvement of 6.03 points across all groups (almost 10% of the maximal range), all strata remained in the severe anxiety range, which highlights how serious anxiety symptoms are in BC patients and how often they persist after overcoming the disease. Analysis of BDI results was more promising, with all groups (except the SG) displaying mild depression symptoms due to scores below ten points. For the Mini-MAC, all results for baseline and post-intervention assessment of constructive and destructive coping styles were average, according to sten norms.

VR proved effective in PA motivation for cancer survivors, who improved their overall activity rating from moderate to high due to the intervention. The DG also showed improved PA levels, though this result was moderate in pre- and post-measures. The mean sleep quality improvement was 1.49 PSQI points, which is 7.1% of the maximum range of the questionnaire. However, since scores above five points indicate poor sleep quality, the therapy only allowed the SG to eliminate sleep disorders.

Even though some parameter changes were insufficient, VR effectiveness should not be discounted since baseline symptoms were high. This interpretation provides a field for further research in which it would be worthwhile investigating whether performing the intervention twice improves its effectiveness or if longer VR exposure (more interventions) has no impact on the tested parameters.

The experimental comparative study design was used following pilot studies and randomised controlled trials previously conducted by the research team (15, 42). The prior studies found that oncological treatment methods and the occurrence of cancer generate so many variables that it is necessary to assess whether the use of VR in some groups will not prove counter-effective. Therefore, it was decided to examine the most numerous groups of patients in BC (in terms of disease stages and treatment) and subject them to a comparative analysis.

The statistical methods used made it possible to confirm the effectiveness of VR (no baseline differences, significant effect of time, non-significant time × group interaction) under various conditions throughout the disease treatment course and determine that there were no contraindications or increased need for VR use among certain groups. Such results suggest that VR can improve BC patient quality of life and psychophysical wellbeing and is effective from diagnosis to the end of the treatment.

Based on the current study, the future focus suggested is justified. Also, against the scientific background of available studies, further research on VR’s effectiveness in improving physical functions seems reasonable. Knowing that VR is a technology that is gaining popularity, it seems that research on a larger sample with more consistent and higher-quality methods is imminent. Furthermore, VR effectiveness should be tested in various health conditions, treatment stages and locations (primary care, hospital, and patient’s homes) to confirm its validity and cost-effectiveness and exclude risks and contraindications.

## Limitations

Despite the promising results, some limitations could not be avoided. The research design did not include a control group, which did not allow for randomization and led to forced stratification. However, this approach was consistent with the assumed aims and addressed the study purpose without affecting the quality of work. Another inherent limitation of VR research is the lack of participant blinding, although every effort was made to ensure that blinding was applied wherever possible, with the clinicians, data collectors, and data analysts blinded during the study. Other unspecified confounding factors may also be considered a limitation. Oncology patients are usually professionally and socially active people. Therefore, we cannot be sure if factors resulting from this type of activity could have influenced the results. The influence of this factors on the results is unknown, but scientific care and the use of standardised measurement tools allow to minimise the margin of error.

Also, the effectiveness of VR vary based on factors such as patient age, treatment stage, symptom severity, session length, and immersion level. VR is not suitable for all patients—contraindications include photosensitive epilepsy, severe motion sickness, balance issues, serious psychiatric disorders, and cognitive impairments. Additionally, side effects like nausea, disorientation, and visual fatigue are more likely during longer or poorly designed sessions, particularly those exceeding 15 minutes (45). According to the results, future research should focus on populations with other medical conditions and use various treatment locations, which may impact efficiency.

## Conclusions

VR is an effective tool for supporting BC patients at different stages of diagnosis and treatment and after healing. Indeed, effectiveness did not differ between patients recently diagnosed, those treated with surgery or undergoing radiation therapy, and patients who had completed treatment. Thus, VR may be recommended as a supportive tool for all BC stages.

## Data Availability

The raw data supporting the conclusions of this article will be made available by the authors, without undue reservation.
